# Unlocking the Potential of Virtual Care in Australian Outpatient Services

**DOI:** 10.5694/mja2.70302

**Published:** 2026-09-17

**Authors:** Ramya Walsan, Rebecca Mitchell, Karol Petrovska, Tracey Webster, Reema Harrison

**Affiliations:** ^1^ Australian Institute of Health Innovation Macquarie University Sydney New South Wales Australia; ^2^ Connected Care, System Performance Support Branch, System Sustainability and Performance Division NSW Ministry of Health Sydney New South Wales Australia; ^3^ Clinical Leadership Effectiveness and Outcomes, Transformation Quality and Safety Division Northern Health Melbourne Victoria Australia

**Keywords:** chronic disease, health planning, public health, public policy, telemedicine

## Abstract

Virtual care has become a critical component of contemporary health system reform, offering opportunities to improve access, continuity and efficiency of care. Virtual care is particularly relevant for chronic disease management, as it can support more continuous, coordinated and person‐centred models of care. This commentary draws on evidence from a national project titled Smarter Hospitals and examines trends in virtual care use across four service areas (cancer, renal disease, mental health or conditions requiring rehabilitation) over 2019–2024, as well as key factors shaping virtual care use and opportunities to realise its full potential.

## 1 Introduction

Despite well‐documented benefits, including improved access, reduced costs and minimised travel burden, particularly for rural or underserved populations [1], virtual care use in Australian outpatient settings has plateaued since the COVID‐19 pandemic, although it remains above pre‐pandemic levels. This stabilisation likely reflects a shift from emergency‐driven uptake to a slower process of embedding virtual care within routine, condition‐appropriate models of care, shaped by funding models and clinician preferences. Growing evidence indicates that, when appropriately integrated and targeted to specific conditions, virtual care can improve outcomes and experiences, particularly in chronic disease management [2].

This article draws on the broader virtual care literature and evidence from a national program of work (the Smarter Hospitals Project) examining virtual care integration and outcomes across four chronic conditions: cancer, renal disease, mental health and conditions requiring rehabilitation in Australia [3]. Key factors shaping current engagement with virtual care and opportunities to realise its full potential are identified and discussed.

## 2 The Vision for Virtual Care in Australia

Virtual care, or telehealth, refers to the delivery of healthcare services through digital platforms [4]. Although traditionally used to support rural and remote communities, the COVID‐19 pandemic accelerated its adoption across healthcare systems. At its peak, virtual care became a critical means of maintaining service access while reducing transmission risk, particularly for people requiring ongoing care, and demonstrated its potential for multidisciplinary management [5].

In response, the Australian Government introduced new Medicare billing items, while governments and health departments invested in digital infrastructure and policy reforms to support virtual care expansion [6]. This progress helped shape a more ambitious national and jurisdictional vision for digitally enabled care. The Australian Digital Health Blueprint outlines a national ambition for a person‐centred, digitally enabled health system [7]. In New South Wales, this is reflected in connected care, signalling a broader move towards integrated, digitally supported patient pathways. Other jurisdictions have similarly aligned their strategies.

## 3 Patterns of Virtual Care Uptake in Australian Outpatient Services

**FIGURE 1:**
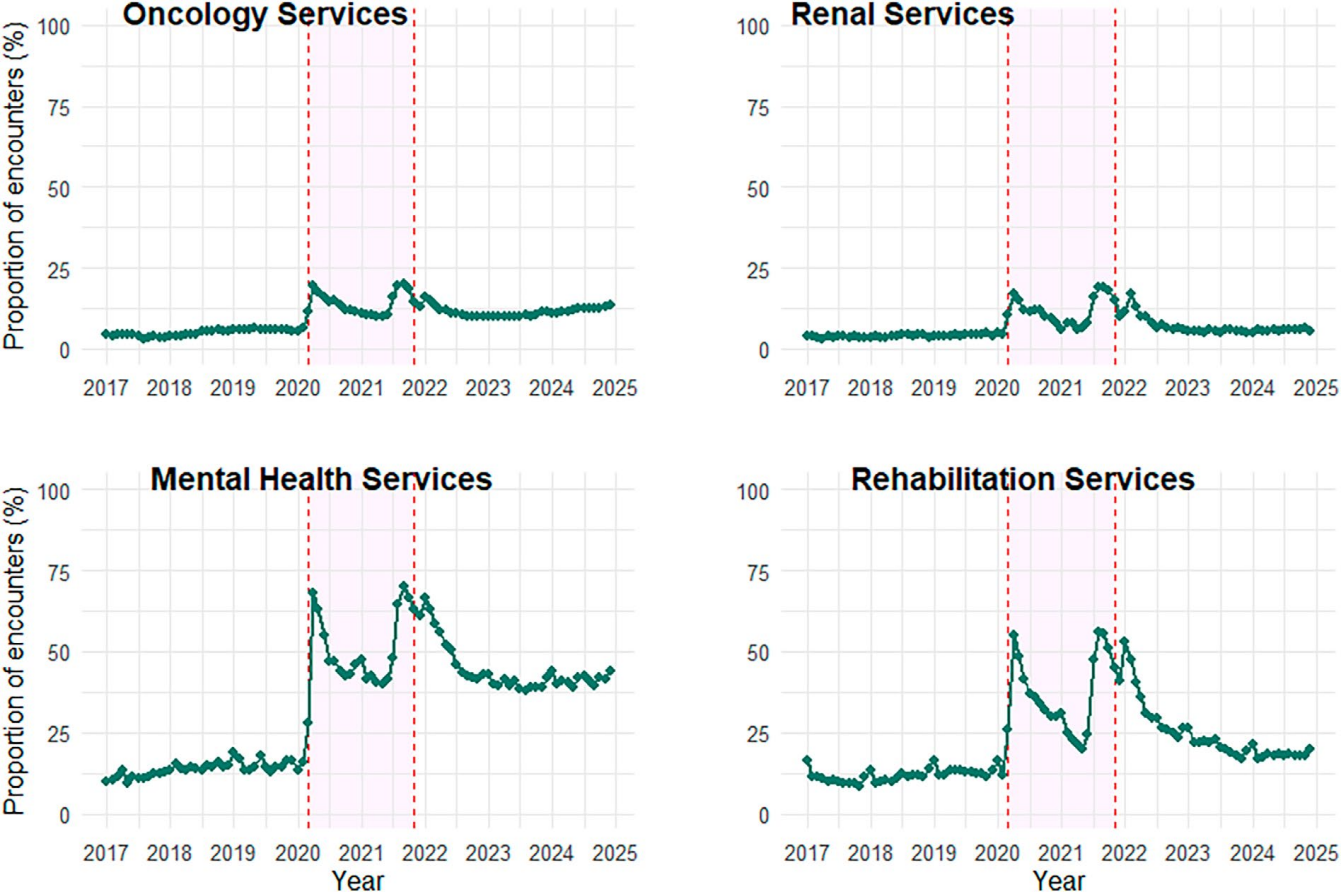
Proportion of included outpatient service events delivered virtually in New South Wales (2019–2024). The *y*‐axis represents the proportion of included outpatient service events delivered virtually. Estimates reflect service‐event modality, not patient‐level virtual care uptake.

Recognising the importance of virtual care in managing chronic conditions, outpatient use data from 2019 to 2024 were examined across four service areas: oncology, renal, mental health and rehabilitative care in New South Wales (Figure 1). Data sources, service definitions, modality classifications and analytic approach are described in Section S1 and Tables S1–S4. Virtual care was defined as consultations delivered by telephone or video. Across the four services, virtual care use increased from pre‐pandemic levels of about 5%–15% to pandemic peaks, ranging from around 20% in oncology and renal services to about 55%–70% in rehabilitation and mental health services. Virtual care use declined after 2021 but remained above pre‐pandemic levels by late 2024, stabilising at around 10%–15% for oncology and renal services, about 20% for rehabilitation and 40%–45% for mental health.

## 4 Slow Progression of Global Efforts to Integrate Virtual Care

Across many countries, national reports show a similar pattern: virtual care use surged during the COVID‐19 pandemic, then declined and stabilised at a higher post‐pandemic baseline. In the United States, virtual care represented less than 1% of outpatient volume before 2020, peaked above 30% during the pandemic and has since stabilised at about 10%–15% [8]. Similar trends are observed in Canada and the United Kingdom, where recent data show virtual care accounting for about 30% of outpatient consultations [9, 10].

## 5 Who Is Using Virtual Care

Data from the Smarter Hospitals Project show that, compared with patients accessing face to face care only, virtual care users are more likely to be younger adults aged 18–44 years (vs. adults aged ≥ 65 years), Australian‐born individuals (vs. overseas‐born), patients with mental or physical health comorbidities (vs. without) and higher users of healthcare (vs. low users) [11]. These patterns align with existing literature: younger adults tend to engage more readily with virtual services [12], individuals with chronic physical or mental health conditions are often early adopters of digitally enabled care [13] and people with greater health system interactions are more likely to use virtual modalities than low‐intensity users.

## 6 Enablers and Opportunities for Delivering High‐Value Virtual Care

To realise the full value of virtual care, health systems need to address four interconnected priorities: (i) re‐designing care models for a digital‐first environment and targeting virtual care models to people who would benefit most; (ii) strengthening digital infrastructure, workforce capability and sustained investment; (iii) embedding robust evaluation to guide continuous improvement; and (iv) reducing digital and structural inequities to ensure virtual care is accessible and effective for all.

### 6.1 Re‐Designing Digitally Enabled Models of Care That Reach Those Who Would Benefit Most

A major opportunity lies in reshaping models of care for a digital‐first environment, rather than adapting traditional, in‐person structures. Many current models were never built with digital delivery in mind and simply replicate face‐to‐face workflows online [14]. When services mirror legacy processes, health systems miss the strengths of digital platforms, including continuous monitoring, proactive intervention and personalised support beyond clinic walls. Importantly, virtual models can extend beyond consultations to support safe treatment delivery, research participation and clinical supervision, including tele‐chemotherapy, tele‐trials, tele‐dialysis and tele‐supervision. This potential needs to be deliberately harnessed to improve access closer to home and strengthen local service capability.

Strengthening virtual care also requires more deliberate service targeting. Rather than prioritising those most likely to benefit, such as people in rural or remote areas, those with mobility challenges or people managing chronic conditions, virtual care is often applied broadly across outpatient populations without sufficient regard for context [15]. This broad application can reduce impact, waste resources and overlook important opportunities to improve equity. Re‐designing care pathways with digital capability and targeted delivery at the centre would allow virtual care to evolve from a supplementary option into a high‐value mode of care.

### 6.2 Strengthening System Capability, Infrastructure and Investment to Support Reliable Virtual Care

Virtual care can only reach its potential when supported by strong digital foundations, a capable workforce and sustained investment [16]. Many health services still lack the core infrastructure required for virtual care, including patient‐facing tools, such as digital front doors, secure messaging, online scheduling, shared care plans and integrated records [17]. Targeted funding to build and maintain digital capabilities would create more connected pathways for patients and clinicians.

Workforce capacity is another critical enabler [18]. Expanding training in virtual care delivery, digital workflows and remote clinical assessment across health services, alongside embedding it in medical, nursing and allied health curricula, would build workforce confidence. Increased investment in innovation and change management is also essential to help services adopt, embed and scale virtual models effectively [19]. Strengthening digital infrastructure, building workforce capability and investing in system‐wide digital health transformation would allow virtual care to function as a core component of service delivery.

### 6.3 Strengthening Evaluation to Support Sustainable Virtual Care

The rapid expansion of virtual care during the COVID‐19 pandemic occurred without clear plans for performance monitoring, outcome measurement or long‐term evaluation, particularly within public systems [20]. As national audits identified, clear metrics or targets were rarely established to assess effectiveness, safety or appropriateness, making it difficult to determine what worked, for whom and under what circumstances [20]. This gap constrains model refinement, clinical confidence and informed funding decisions. Building sustainability requires robust evaluation frameworks embedded from the outset of virtual care reforms, with clearly defined metrics and agreed outcomes. High‐quality research, supported by consistent data collection and transparent reporting, would enable continuous improvement and strengthen the evidence base across diverse populations and services. This, in turn, would support the scaling of effective models and help shift virtual care from a reactive measure to an evidence‐informed component of healthcare.

### 6.4 Addressing Inequities to Improve Access and Engagement

For virtual care to deliver equitable patient outcomes, it must be accessible to communities facing the greatest barriers to digital participation. People experiencing homelessness, living on low incomes or residing in remote areas often lack reliable devices or internet access [21], while nearly 2.8 million Australians are digitally excluded and around half of remote Indigenous communities lack mobile coverage [22]. Addressing these gaps requires targeted investment in digital infrastructure, device access through public libraries and community centres and affordable connectivity. Much of this lies beyond the direct remit of health systems and requires coordinated action across government and community sectors.

Older adults, culturally and linguistically diverse populations and socio‐economically disadvantaged groups also face barriers related to digital literacy, low confidence using technology, language and cultural preferences [23, 24]. Strengthening interpreter support, tailoring platforms to diverse language needs and offering hands‐on digital literacy coaching can improve engagement and trust in virtual healthcare. Realising the equity potential also requires moving beyond short‐term project‐based implementation towards programmatic approaches that embed models with clear ownership, co‐design and monitoring mechanisms [25].

## 7 Conclusion

Virtual care has matured from an emergency response into a core component of contemporary outpatient delivery. To fully realise its potential in Australia, a more strategic, equitable and evidence‐informed approach is essential. Stabilisation in use reflects not a decline in interest, but the need for care models to be intentionally designed for digital delivery and supported by robust digital infrastructure, a skilled workforce and sustained investment. Strengthening evaluation and continuous improvement and addressing inequities will ensure that virtual care expands access rather than reinforcing existing care gaps.

## Author Contributions

**Ramya Walsan:** conceptualisation, data curation, methodology, formal analysis, validation, visualisation, software, writing – original draft, writing – review and editing. **Rebecca Mitchell:** methodology, supervision, validation, writing – review and editing. **Karol Petrovska:** writing – review and editing. **Tracey Webster:** writing – review and editing. **Reema Harrison:** conceptualisation, funding acquisition, supervision, resources, validation, writing – review and editing.

## Funding

Reema Harrison received funding from the National Health and Medical Research Council Partnership Scheme (grant no. 2015544) for the project Smarter Hospitals. The funder was not involved in the study design, data collection, analysis, interpretation or the writing of the manuscript.

## Disclosure

Not commissioned; externally peer reviewed.

## Conflicts of Interest

The authors declare no conflicts of interest.

## Supporting information


**Section S1**. Methods for Figure 1: Proportion of virtual and in‐person care modalities, 2019–2024.
**Table S1:** Proportion of virtual and in‐person consultations in oncology services by month in New South Wales (2017–2024).
**Table S2:** Proportion of virtual and in‐person consultations in renal services by month in New South Wales (2017–2024).
**Table S3:** Proportion of virtual and in‐person consultations in mental health services by month in New South Wales (2017–2024).
**Table S4:** Proportion of virtual and in‐person consultations in rehabilitation services by month in New South Wales (2017–2024).

## Data Availability

The data that support the findings of this study are available from the New South Wales Department of Health. Restrictions apply to the availability of these data, which were used under licence for the current study, so are not publicly available.

## References

[1] J. J. Moffatt and D. S. Eley , “The Reported Benefits of Telehealth for Rural Australians,” Australian Health Review 34, no. 3 (2010): 276–281. 10.1071/AH09794 20797357

[2] Y. Ma , C. Zhao , Y. Zhao , et al., “Telemedicine Application in Patients With Chronic Disease: A Systematic Review and Meta‐Analysis,” BMC Medical Informatics and Decision Making 22, no. 1 (2022): 105. 10.1186/s12911-022-01845-2 PMC9017076 35440082

[3] R. Harrison , R. Mitchell , R. Walsan , et al., “Unlocking the Promise of Virtual Care in Hospitals: The Smarter Hospitals Project Protocol,” BMC Health Services Research 25 (2025): 1058. 10.1186/s12913-025-13129-2 PMC12337380 40790486

[4] A. Haleem , M. Javaid , R. P. Singh , and R. Suman , “Telemedicine for Healthcare: Capabilities, Features, Barriers, and Applications,” Sensors International 2 (2021): 100117. 10.1016/j.sintl.2021.100117 PMC8590973 34806053

[5] D. Rodin , M. Lovas , and A. Berlin , “The Reality of Virtual Care: Implications for Cancer Care Beyond the Pandemic,” Healthcare 8, no. 4 (2020): 100480. 10.1016/j.hjdsi.2020.100480 PMC7578846 33129178

[6] Australian Government Department of Health and Aged Care , “Telehealth,” (2025), accessed November 2, 2025, https://www.health.gov.au/topics/health‐technologies‐and‐digital‐health/about/telehealth.

[7] Australian Government Department of Health and Aged Care, The Digital Health Blueprint and Action Plan 2023–2033 (Australian Government, 2022), accessed November 2, 2025, https://www.health.gov.au/resources/publications/the‐digital‐health‐blueprint‐and‐action‐plan‐2023‐2033.

[8] American Hospital Association , “Telehealth Fact Sheet,” (2025), accessed November 1, 2025, https://www.aha.org/fact‐sheets/2025‐02‐07‐fact‐sheet‐telehealth.

[9] Government of Canada , Virtual Care Policy Framework (Health Canada, 2021), accessed November 1, 2025, https://www.canada.ca/en/health‐canada/corporate/transparency/health‐agreements/bilateral‐agreement‐pan‐canadian‐virtual‐care‐priorities‐covid‐19/policy‐framework.html.

[10] R. Lewis , J. Smith , R. Posaner , et al., “Learning from the Pandemic Shift of Outpatient Services to a Remote Footing: A Rapid Evaluation Study,” (2023), accessed September 7, 2026, https://birminghamhealthpartners.co.uk/wp‐content/uploads/2024/01/Learning‐from‐the‐pandemic‐shift‐of‐outpatient‐services‐to‐a‐remote‐footing‐a‐BHP‐rapid‐evaluation‐study.pdf.

[11] R. Walsan , R. Harrison , J. Westbrook , et al., “Characteristics of Patients Accessing Outpatient Oncology Services Virtually and Predictors of Subsequent Unplanned Emergency Department Presentations in 78,323 Adults in Australia: Retrospective Cohort Study,” Journal of Medical Internet Research 28 (2026): e87694. 10.2196/87694 PMC13132016 42060635

[12] S. K. Mistry , M. Shaw , F. Raffan , et al., “Inequity in Access and Delivery of Virtual Care Interventions: A Scoping Review,” International Journal of Environmental Research and Public Health 19, no. 15 (2022): 9411. 10.3390/ijerph19159411 PMC9367842 35954768

[13] K. Ward , S. Vagholkar , J. Lane , S. Raghuraman , and A. Y. S. Lau , “Are Chronic Condition Management Visits Translatable to Telehealth? Analysis of In‐Person Consultations in Primary Care,” International Journal of Medical Informatics 178 (2023): 105197. 10.1016/j.ijmedinf.2023.105197 37619394

[14] I. J. Borges do Nascimento , H. Abdulazeem , L. T. Vasanthan , et al., “Barriers and Facilitators to Utilizing Digital Health Technologies by Healthcare Professionals,” npj Digital Medicine 6, no. 1 (2023): 161. 10.1038/s41746-023-00899-4 PMC10507089 37723240

[15] S. Osman , K. Churruca , L. A. Ellis , and J. Braithwaite , “Beyond the Planned and Expected: The Unintended Consequences of Telehealth in Rural and Remote Australia Through a Complexity Lens,” Medical Journal of Australia 220, no. 10 (2024): 496–498, https://www.mja.com.au/journal/2024/220/10/beyond‐planned‐and‐expected‐unintended‐consequences‐telehealth‐rural‐and‐remote. 10.5694/mja2.52294 38703008

[16] J. Jonnagaddala , M. A. Godinho , and S. T. Liaw , “From Telehealth to Virtual Primary Care in Australia? A Rapid Scoping Review,” International Journal of Medical Informatics 151 (2021): 104470. 10.1016/j.ijmedinf.2021.104470 PMC9761082 34000481

[17] Australian Government Productivity Commission , Leveraging Digital Technology in Healthcare (Australian Government, 2024), accessed November 2, 2025, https://assets.pc.gov.au/research/completed/digital‐healthcare/digital‐healthcare.pdf.

[18] Australian Digital Health Agency , Australian Digital Health Capability Framework (Australian Government, 2017), accessed November 7, 2025, https://digitalhealthworkforce.org.au/wp‐content/uploads/2024/08/Australian‐Digital‐Health‐Capability‐Framework‐V1.2.pdf.

[19] R. Harrison , M. Prokopy , and T. Perreira , “Virtual Care Post‐Pandemic: Why User Engagement Is Critical to Create and Optimise Future Models of Care,” DIGITAL HEALTH 8 (2022): 20552076221131455. 10.1177/20552076221131455 PMC9551327 36238755

[20] Australian National Audit Office , Performance Audit Report No. 10 2022–23: Expansion of Telehealth Services (ANAO, 2023), accessed November 2, 2025, https://www.anao.gov.au/sites/default/files/2023‐01/Auditor‐General_Report_2022‐23_10.pdf.

[21] K. Fisher and P. Magin , “The Telehealth Divide: Health Inequity During the COVID‐19 Pandemic,” Family Practice 39, no. 3 (2021): 547–549. 10.1093/fampra/cmab173 PMC9383174 34964886

[22] E. Parke , ”Digital Divide Report Shows Thousands of Australians in Remote Communities Still Don't Have Internet Access,” ABC News, (2024), accessed 3 August 2026, https://www.abc.net.au/news/2024‐12‐10/digital‐divide‐report‐remote‐communities‐internet‐access/104694460.

[23] S. Mathew , M. S. Fitts , Z. Liddle , et al., “Telehealth in Remote Australia: A Supplementary Tool or an Alternative Model of Care Replacing Face‐To‐Face Consultations?,” BMC Health Services Research 23, no. 1 (2023): 341. 10.1186/s12913-023-09265-2 PMC10074370 37020234

[24] R. B. Khatri and Y. Assefa , “Access to Health Services Among Culturally and Linguistically Diverse Populations in the Australian Universal Health Care System: Issues and Challenges,” BMC Public Health 22, no. 1 (2022): 880. 10.1186/s12889-022-13256-z PMC9063872 35505307

[25] J. Shaw , L. C. Brewer , and T. Veinot , “Recommendations for Health Equity and Virtual Care Arising From the COVID‐19 Pandemic: Narrative Review,” JMIR Formative Research 5, no. 4 (2021): e23233. 10.2196/23233 PMC8023377 33739931

