# Supplementary material for: Unlocking the Potential of Virtual Care in Australian Outpatient Services
Source: Med J Aust. 2026 Sep 17;224(9):e70302. doi: 10.5694/mja2.70302 (PMC13585566; doi:10.5694/mja2.70302)
Supplement: Supplementary file 1 [file MJA2-224-0-s001.pdf]

Supporting Information

Supplementary material

This appendix was part of the submitted manuscript and has been peer reviewed. It is posted as supplied by the authors.

Appendix to: R. Walsan, R. Mitchell, K. Petrovska, et al. Unlocking the Potential of Virtual Care in Australian Outpatient Services. *Medical Journal of Australia* 2026; doi: 10.5694/mja2.70302.

SECTION S1. Methods for Figure 1: Proportion of virtual and in-person care modalities, 2019–2024

This Supporting Information file provides a summary of the methods used to generate Figure 1 in the manuscript. Figure 1 presents the proportion of included outpatient service events delivered virtually across oncology, renal disease, mental health, and conditions requiring rehabilitation in New South Wales from 2019 to 2024.

Data Sources

Outpatient data were obtained from the NSW Non-Admitted Patient Data Collection. These datasets capture public outpatient services in New South Wales, including patient demographics, service dates, service types, contact streams, and modes of care delivery. The analysis included outpatient service records for 3,365,406 individuals in New South Wales from 2019 to 2024, across oncology, renal disease, mental health, and conditions requiring rehabilitation.

Outpatient service groups were identified using the TIER 2 Non-Admitted Service Classification. Oncology services included medical oncology consultation, radiation therapy consultation, chemotherapy treatment, radiation therapy treatment, and radiation therapy simulation and planning. Renal services included nephrology consultations and hospital-delivered renal dialysis. Mental health services included psychiatry, psychogeriatric services, psychology, general counselling, and specialist mental health services. Rehabilitation services included general rehabilitation, cardiac rehabilitation, and pulmonary rehabilitation.

Classification of care modality

Services were classified as either virtual care or in-person care based on the mode-of-delivery variable in the outpatient dataset. Virtual care was defined as consultations delivered via telephone or video.

Analysis

For each month, the number of services delivered virtually was calculated as the number of virtual service events divided by the total number of included service events, multiplied by 100. This monthly proportion is presented on the vertical (y) axis of Figure 1. Because included service events were classified as either virtual or in-person, the remaining proportion represents in-person service events. Proportions were calculated over time to examine changes in virtual care use in New South Wales between 2019 and 2024. The major COVID-19 lockdown period in New South Wales, from 1 March 2020 to 31 July 2021, was highlighted in the figure to provide context for changes in virtual care delivery during this period.

Figure generation

Figure 1 was generated using R software, version 4.2.2.

Limitations of Figure 1

Figure 1 provides descriptive context on recorded outpatient service modality trends in NSW. The observed plateau refers to the proportion of outpatient service events delivered virtually not to stability in total outpatient activity, virtual care volume, or demand. The estimates are based on service events rather than individual patients, so patients with multiple outpatient encounters may contribute more than once to the monthly proportions. The analysis does not assess appropriateness, patient outcomes, safety, access, rurality, socioeconomic status, age, language, digital exclusion, clinical acuity, or patient preference, and should not be interpreted as direct evidence about which patients or models of care benefit most from virtual care or whether virtual care affects inequities. Figure 1 reflects recorded telephone or video outpatient consultations only and does not capture other virtual care models, such as remote monitoring, connected care, or other digitally enabled models of care. These broader aspects are considered

in the commentary, which draws on the wider Smarter Hospitals Project and existing evidence base.

TABLE S1: Proportion of virtual and in-person consultations in oncology services by month in New South Wales (2017-2024)

Year	Month	In person consultations	Virtual consultations	Total consultations	Proportion of virtual encounters	Proportion of in person encounters
2017	Jan	72675	3398	76073	4.5%	95.5%
	Feb	77935	3265	81200	4.0%	96.0%
	Mar	78838	3717	82555	4.5%	95.5%
	April	69942	3379	73321	4.6%	95.4%
	May	87702	4113	91815	4.5%	95.5%
	June	78257	3676	81933	4.5%	95.5%
	July	79064	3017	82081	3.7%	96.3%
	Aug	88542	2876	91418	3.1%	96.9%
	Sep	80773	2875	83648	3.4%	96.6%
	Oct	78899	3096	81995	3.8%	96.2%
	Nov	87197	3176	90373	3.5%	96.5%
	Dec	71583	2618	74201	3.5%	96.5%
2018	Jan	80414	3208	83622	3.8%	96.2%
	Feb	79635	3032	82667	3.7%	96.3%
	Mar	84330	3567	87897	4.1%	95.9%
	April	75937	3326	79263	4.2%	95.8%
	May	88539	3860	92399	4.2%	95.8%
	June	77178	3395	80573	4.2%	95.8%
	July	148008	8093	156101	5.2%	94.8%
	Aug	142956	8193	151149	5.4%	94.6%
	Sep	124887	6961	131848	5.3%	94.7%
	Oct	128248	7840	136088	5.8%	94.2%
	Nov	130731	7825	138556	5.6%	94.4%
	Dec	107162	6338	113500	5.6%	94.4%
	Jan	115236	7198	122434	5.9%	94.1%
	Feb	116405	7396	123801	6.0%	94.0%

	Mar	117207	7658	124865	6.1%	93.9%
	April	113578	7309	120887	6.0%	94.0%
2019	May	132379	8841	141220	6.3%	93.7%
	June	114831	7196	122027	5.9%	94.1%
	July	143567	9079	152646	5.9%	94.1%
	Aug	136033	8670	144703	6.0%	94.0%
	Sep	129492	8152	137644	5.9%	94.1%
	Oct	141149	8693	149842	5.8%	94.2%
	Nov	135565	8355	143920	5.8%	94.2%
	Dec	122392	7199	129591	5.6%	94.4%
2020	Jan	131725	7871	139596	5.6%	94.4%
	Feb	130779	8668	139447	6.2%	93.8%
	Mar	123585	15713	139298	11.3%	88.7%
	April	99225	23795	123020	19.3%	80.7%
	May	104885	22108	126993	17.4%	82.6%
	June	111638	21129	132767	15.9%	84.1%
	July	120765	20342	141107	14.4%	85.6%
	Aug	117801	20407	138208	14.8%	85.2%
	Sep	129207	20128	149335	13.5%	86.5%
	Oct	125715	17491	143206	12.2%	87.8%
	Nov	126403	16997	143400	11.9%	88.1%
	Dec	119088	15061	134149	11.2%	88.8%
2021	202101	109751	13324	123075	10.8%	89.2%
	202102	122418	14418	136836	10.5%	89.5%
	202103	140105	16628	156733	10.6%	89.4%
	202104	124473	13506	137979	9.8%	90.2%
	202105	130879	14190	145069	9.8%	90.2%
	202106	129500	15039	144539	10.4%	89.6%
	202107	121065	23068	144133	16.0%	84.0%
	202108	120189	29514	149703	19.7%	80.3%
	202109	117375	29676	147051	20.2%	79.8%
	202110	110116	24606	134722	18.3%	81.7%
	202111	127327	21777	149104	14.6%	85.4%

	202112	116177	17127	133304	12.8%	87.2%
	Jan	100035	18876	118911	15.9%	84.1%
	Feb	117884	20614	138498	14.9%	85.1%
2022	Mar	135883	20816	156699	13.3%	86.7%
	April	114206	15518	129724	12.0%	88.0%
	May	128140	17112	145252	11.8%	88.2%
	June	123902	15401	139303	11.1%	88.9%
	July	126375	15408	141783	10.9%	89.1%
	Aug	145732	16734	162466	10.3%	89.7%
	Sep	137349	15207	152556	10.0%	90.0%
	Oct	130724	14477	145201	10.0%	90.0%
	Nov	146200	16504	162704	10.1%	89.9%
	Dec	124455	13732	138187	9.9%	90.1%
2023	Jan	127850	13750	141600	9.7%	90.3%
	Feb	132726	14818	147544	10.0%	90.0%
	Mar	153462	16803	170265	9.9%	90.1%
	April	119413	13313	132726	10.0%	90.0%
	May	155018	16861	171879	9.8%	90.2%
	June	137554	15126	152680	9.9%	90.1%
	July	131302	14802	146104	10.1%	89.9%
	Aug	147022	16797	163819	10.3%	89.7%
	Sep	132700	14970	147670	10.1%	89.9%
	Oct	135823	15447	151270	10.2%	89.8%
	Nov	130535	16646	147181	11.3%	88.7%
	Dec	107675	13678	121353	11.3%	88.7%
2024	Jan	121500	14699	136199	10.8%	89.2%
	Feb	126985	15829	142814	11.1%	88.9%
	Mar	116450	15342	131792	11.6%	88.4%
	April	119658	15478	135136	11.5%	88.5%
	May	131752	17944	149696	12.0%	88.0%
	June	110903	15768	126671	12.4%	87.6%
	July	137642	19829	157471	12.6%	87.4%
	Aug	133863	19412	153275	12.7%	87.3%

	Sep	128443	18653	147096	12.7%	87.3%
	Oct	133817	18754	152571	12.3%	87.7%
	Nov	119953	17748	137701	12.9%	87.1%
	Dec	101916	15792	117708	13.4%	86.6%

TABLE S2: Proportion of virtual and in-person consultations in renal services by month in New South Wales (2017-2024)

Year	Month	In person consultations	Virtual consultations	Total consultations	Proportion of virtual encounters	Proportion of in person encounters
2017	Jan	26345	1079	27424	3.9%	96.1%
	Feb	28637	1225	29862	4.1%	95.9%
	Mar	35136	1276	36412	3.5%	96.5%
	April	30124	961	31085	3.1%	96.9%
	May	34474	1318	35792	3.7%	96.3%
	June	31669	1194	32863	3.6%	96.4%
	July	34473	1308	35781	3.7%	96.3%
	Aug	35993	1479	37472	3.9%	96.1%
	Sep	33741	1262	35003	3.6%	96.4%
	Oct	35183	1370	36553	3.7%	96.3%
	Nov	36222	1363	37585	3.6%	96.4%
	Dec	33474	1141	34615	3.3%	96.7%
2018	Jan	32569	1129	33698	3.4%	96.6%
	Feb	33332	1294	34626	3.7%	96.3%
	Mar	36293	1373	37666	3.6%	96.4%
	April	34758	1249	36007	3.5%	96.5%
	May	37234	1513	38747	3.9%	96.1%
	June	35658	1505	37163	4.0%	96.0%
	July	49567	2148	51715	4.2%	95.8%
	Aug	51289	2247	53536	4.2%	95.8%
	Sep	47717	1885	49602	3.8%	96.2%
	Oct	46957	2083	49040	4.2%	95.8%
	Nov	47677	2100	49777	4.2%	95.8%
	Dec	44948	1687	46635	3.6%	96.4%
	Jan	43247	1869	45116	4.1%	95.9%
	Feb	44349	1903	46252	4.1%	95.9%

2019	Mar	47831	2012	49843	4.0%	96.0%
	April	44216	1897	46113	4.1%	95.9%
	May	48725	2157	50882	4.2%	95.8%
	June	42947	1813	44760	4.1%	95.9%
	July	46641	2158	48799	4.4%	95.6%
	Aug	47639	2183	49822	4.4%	95.6%
	Sep	45716	2203	47919	4.6%	95.4%
	Oct	47842	2208	50050	4.4%	95.6%
	Nov	46250	2287	48537	4.7%	95.3%
	Dec	45878	1943	47821	4.1%	95.9%
2020	Jan	44085	2188	46273	4.7%	95.3%
	Feb	46903	2047	48950	4.2%	95.8%
	Mar	45123	5107	50230	10.2%	89.8%
	April	39697	8238	47935	17.2%	82.8%
	May	42684	7648	50332	15.2%	84.8%
	June	43744	5900	49644	11.9%	88.1%
	July	45696	5883	51579	11.4%	88.6%
	Aug	44662	6143	50805	12.1%	87.9%
	Sep	44848	6129	50977	12.0%	88.0%
	Oct	46264	5009	51273	9.8%	90.2%
	Nov	46595	4898	51493	9.5%	90.5%
	Dec	46724	3971	50695	7.8%	92.2%
2021	Jan	42694	2683	45377	5.9%	94.1%
	Feb	44222	3918	48140	8.1%	91.9%
	Mar	48215	4175	52390	8.0%	92.0%
	April	45037	2753	47790	5.8%	94.2%
	May	46455	3258	49713	6.6%	93.4%
	June	44416	3695	48111	7.7%	92.3%
	July	40190	7697	47887	16.1%	83.9%
	Aug	39300	9336	48636	19.2%	80.8%
	Sep	37796	8783	46579	18.9%	81.1%
	Oct	37816	8278	46094	18.0%	82.0%
	Nov	42218	7389	49607	14.9%	85.1%

	Dec	42336	4776	47112	10.1%	89.9%
2022	Jan	38515	4941	43456	11.4%	88.6%
	Feb	37704	7738	45442	17.0%	83.0%
	Mar	43643	6512	50155	13.0%	87.0%
	April	40227	4351	44578	9.8%	90.2%
	May	44607	4832	49439	9.8%	90.2%
	June	42760	3638	46398	7.8%	92.2%
	July	42960	2940	45900	6.4%	93.6%
	Aug	44071	3449	47520	7.3%	92.7%
	Sep	42461	2874	45335	6.3%	93.7%
	Oct	42875	2775	45650	6.1%	93.9%
	Nov	43299	2912	46211	6.3%	93.7%
	Dec	41747	2542	44289	5.7%	94.3%
2023	Jan	40025	2255	42280	5.3%	94.7%
	Feb	42379	2486	44865	5.5%	94.5%
	Mar	47724	2661	50385	5.3%	94.7%
	April	42337	2083	44420	4.7%	95.3%
	May	44246	2838	47084	6.0%	94.0%
	June	40616	2400	43016	5.6%	94.4%
	July	44629	2252	46881	4.8%	95.2%
	Aug	46465	2843	49308	5.8%	94.2%
	Sep	44173	2829	47002	6.0%	94.0%
	Oct	44970	2620	47590	5.5%	94.5%
	Nov	45156	2655	47811	5.6%	94.4%
	Dec	43399	2247	45646	4.9%	95.1%
2024	Jan	43421	2332	45753	5.1%	94.9%
	Feb	44242	2701	46943	5.8%	94.2%
	Mar	45749	2722	48471	5.6%	94.4%
	April	43300	2518	45818	5.5%	94.5%
	May	47636	2929	50565	5.8%	94.2%
	June	43811	2387	46198	5.2%	94.8%
	July	46830	2903	49733	5.8%	94.2%
	Aug	47074	2927	50001	5.9%	94.1%

	Sep	42829	2592	45421	5.7%	94.3%
	Oct	44345	2896	47241	6.1%	93.9%
	Nov	44255	2972	47227	6.3%	93.7%
	Dec	41836	2460	44296	5.6%	94.4%

TABLE S3: Proportion of virtual and in-person consultations in mental health services by month in New South Wales (2017-2024)

Year	Month	In person consultations	Virtual consultations	Total consultations	Proportion of virtual encounters	Proportion of in person encounters
2017	Jan	14890	1581	16471	9.6%	90.4%
	Feb	16822	1911	18733	10.2%	89.8%
	Mar	19798	2572	22370	11.5%	88.5%
	April	15735	2372	18107	13.1%	86.9%
	May	21212	2216	23428	9.5%	90.5%
	June	19045	2456	21501	11.4%	88.6%
	July	17540	2156	19696	10.9%	89.1%
	Aug	19640	2394	22034	10.9%	89.1%
	Sep	17182	2188	19370	11.3%	88.7%
	Oct	18167	2568	20735	12.3%	87.7%
	Nov	18329	2618	20947	12.5%	87.5%
	Dec	14068	2046	16114	12.7%	87.3%
2018	Jan	15688	2456	18144	13.5%	86.5%
	Feb	16076	2861	18937	15.1%	84.9%
	Mar	16748	2714	19462	13.9%	86.1%
	April	14984	2331	17315	13.5%	86.5%
	May	17447	2957	20404	14.5%	85.5%
	June	15459	2460	17919	13.7%	86.3%
	July	16532	2529	19061	13.3%	86.7%
	Aug	16998	2976	19974	14.9%	85.1%
	Sep	15301	2552	17853	14.3%	85.7%
	Oct	15896	3032	18928	16.0%	84%
	Nov	15869	2642	18511	14.3%	85.7%
	Dec	12141	2123	14264	14.9%	85.1%
2019	Jan	12720	2914	15634	18.6%	81.4%
	Feb	14088	2802	16890	16.6%	83.4%

	Mar	15820	2416	18236	13.2%	86.8%
	April	14704	2261	16965	13.3%	86.7%
	May	17013	2785	19798	14.1%	85.9%
	June	13361	2885	16246	17.8%	82.2%
	July	17160	2882	20042	14.4%	85.6%
	Aug	16725	2487	19212	12.9%	87.1%
	Sep	16068	2733	18801	14.5%	85.5%
	Oct	16973	2870	19843	14.5%	85.5%
	Nov	14987	2921	17908	16.3%	83.7%
	Dec	12518	2397	14915	16.1%	83.9%
2020	Jan	14603	2192	16795	13.1%	86.9%
	Feb	14376	2686	17062	15.7%	84.3%
	Mar	12850	4944	17794	27.8%	72.2%
	April	4990	10631	15621	68.1%	31.9%
	May	5684	9722	15406	63.1%	36.9%
	June	7217	8856	16073	55.1%	44.9%
	July	10569	9414	19983	47.1%	52.9%
	Aug	10259	9108	19367	47.0%	53.0%
	Sep	11673	9256	20929	44.2%	55.8%
	Oct	11630	8648	20278	42.6%	57.4%
	Nov	10681	8092	18773	43.1%	56.9%
	Dec	8561	7362	15923	46.2%	53.8%
2021	Jan	7412	6743	14155	47.6%	52.4%
	Feb	9819	6933	16752	41.4%	58.6%
	Mar	10951	8116	19067	42.6%	57.4%
	April	9749	6549	16298	40.2%	59.8%
	May	10145	6745	16890	39.9%	60.1%
	June	9758	6969	16727	41.7%	58.3%
	July	8737	8013	16750	47.8%	52.2%
	Aug	5614	10286	15900	64.7%	35.3%
	Sep	4598	10845	15443	70.2%	29.8%
	Oct	4782	9589	14371	66.7%	33.3%
	Nov	5642	9612	15254	63.0%	37.0%

	Dec	5225	8254	13479	61.2%	38.8%
2022	Jan	3974	7899	11873	66.5%	33.5%
	Feb	5166	8731	13897	62.8%	37.2%
	Mar	6715	9519	16234	58.6%	41.4%
	April	5416	6886	12302	56.0%	44.0%
	May	7075	7652	14727	52.0%	48.0%
	June	6547	6726	13273	50.7%	49.3%
	July	8595	7375	15970	46.2%	53.8%
	Aug	10461	8013	18474	43.4%	56.6%
	Sep	10167	7489	17656	42.4%	57.6%
	Oct	9895	7192	17087	42.1%	57.9%
	Nov	10757	7639	18396	41.5%	58.5%
	Dec	8563	6519	15082	43.2%	56.8%
2023	Jan	8516	6410	14926	42.9%	57.1%
	Feb	10001	6621	16622	39.8%	60.2%
	Mar	11745	7678	19423	39.5%	60.5%
	April	8821	6190	15011	41.2%	58.8%
	May	11178	7332	18510	39.6%	60.4%
	June	9785	6756	16541	40.8%	59.2%
	July	10120	6269	16389	38.3%	61.7%
	Aug	11513	6992	18505	37.8%	62.2%
	Sep	10030	6350	16380	38.8%	61.2%
	Oct	10304	6617	16921	39.1%	60.9%
	Nov	10614	6771	17385	38.9%	61.1%
	Dec	8137	5911	14048	42.1%	57.9%
2024	Jan	8379	6572	14951	44.0%	56.0%
	Feb	10087	6739	16826	40.1%	59.9%
	Mar	9805	6786	16591	40.9%	59.1%
	April	9654	6551	16205	40.4%	59.6%
	May	11505	7292	18797	38.8%	61.2%
	June	8678	6314	14992	42.1%	57.9%
	July	10330	7606	17936	42.4%	57.6%
	Aug	10063	7028	17091	41.1%	58.9%

	Sep	9542	6153	15695	39.2%	60.8%
	Oct	10051	7228	17279	41.8%	58.2%
	Nov	9696	6839	16535	41.4%	58.6%
	Dec	7741	6075	13816	44.0%	56.0%

TABLE S4: Proportion of virtual and in-person consultations in rehabilitation services by month in New South Wales (2017-2024)

Year	Month	In person consultations	Virtual consultations	Total consultations	Proportion of virtual encounters	Proportion of in person encounters
2017	Jan	16313	3195	19508	16.4%	83.6%
	Feb	26425	3424	29849	11.5%	88.5%
	Mar	31333	3911	35244	11.1%	88.9%
	April	21000	2531	23531	10.8%	89.2%
	May	28924	3113	32037	9.7%	90.3%
	June	26613	3058	29671	10.3%	89.7%
	July	27531	2955	30486	9.7%	90.3%
	Aug	32429	3329	35758	9.3%	90.7%
	Sep	27023	2813	29836	9.4%	90.6%
	Oct	27997	2800	30797	9.1%	90.9%
	Nov	30011	2727	32738	8.3%	91.7%
	Dec	18846	2356	21202	11.1%	88.9%
2018	Jan	18673	2911	21584	13.5%	86.5%
	Feb	28093	2919	31012	9.4%	90.6%
	Mar	30387	3333	33720	9.9%	90.1%
	April	25149	2869	28018	10.2%	89.8%
	May	31297	3348	34645	9.7%	90.3%
	June	25908	3117	29025	10.7%	89.3%
	July	39718	5636	45354	12.4%	87.6%
	Aug	43380	5599	48979	11.4%	88.6%
	Sep	37640	4925	42565	11.6%	88.4%
	Oct	40014	5473	45487	12.0%	88.0%
	Nov	41095	5243	46338	11.3%	88.7%
	Dec	25760	4099	29859	13.7%	86.3%
2019	Jan	26553	5103	31656	16.1%	83.9%
	Feb	38032	5150	43182	11.9%	88.1%

	Mar	40040	5479	45519	12.0%	88.0%
	April	33981	5288	39269	13.5%	86.5%
	May	42403	6407	48810	13.1%	86.9%
	June	34258	5174	39432	13.1%	86.9%
	July	43752	6389	50141	12.7%	87.3%
	Aug	43592	6404	49996	12.8%	87.2%
	Sep	42089	5950	48039	12.4%	87.6%
	Oct	43731	6267	49998	12.5%	87.5%
	Nov	41414	5360	46774	11.5%	88.5%
	Dec	28358	4264	32622	13.1%	86.9%
2020	Jan	27467	5364	32831	16.3%	83.7%
	Feb	40591	5301	45892	11.6%	88.4%
	Mar	30053	10634	40687	26.1%	73.9%
	April	13107	15859	28966	54.8%	45.2%
	May	16192	15109	31301	48.3%	51.7%
	June	19463	13626	33089	41.2%	58.8%
	July	23824	13877	37701	36.8%	63.2%
	Aug	23471	13043	36514	35.7%	64.3%
	Sep	25776	13113	38889	33.7%	66.3%
	Oct	24108	11374	35482	32.1%	67.9%
	Nov	26173	11167	37340	29.9%	70.1%
	Dec	21153	8941	30094	29.7%	70.3%
2021	Jan	16892	7579	24471	31.0%	69.0%
	Feb	27286	9026	36312	24.9%	75.1%
	Mar	33216	9829	43045	22.8%	77.2%
	April	29392	8062	37454	21.5%	78.5%
	May	32611	8017	40628	19.7%	80.3%
	June	29395	9366	38761	24.2%	75.8%
	July	17438	15884	33322	47.7%	52.3%
	Aug	14457	18454	32911	56.1%	43.9%
	Sep	13998	17456	31454	55.5%	44.5%
	Oct	14157	14792	28949	51.1%	48.9%
	Nov	18397	15156	33553	45.2%	54.8%

	Dec	15886	10971	26857	40.8%	59.2%
2022	Jan	9200	10358	19558	53.0%	47.0%
	Feb	14111	12867	26978	47.7%	52.3%
	Mar	20260	13765	34025	40.5%	59.5%
	April	17591	9775	27366	35.7%	64.3%
	May	24573	10916	35489	30.8%	69.2%
	June	22613	9412	32025	29.4%	70.6%
	July	24126	10076	34202	29.5%	70.5%
	Aug	30303	10876	41179	26.4%	73.6%
	Sep	27988	9672	37660	25.7%	74.3%
	Oct	27727	9101	36828	24.7%	75.3%
	Nov	32558	10065	42623	23.6%	76.4%
	Dec	21616	7775	29391	26.5%	73.5%
2023	Jan	20058	7124	27182	26.2%	73.8%
	Feb	29813	8314	38127	21.8%	78.2%
	Mar	35116	9913	45029	22.0%	78.0%
	April	25110	7309	32419	22.5%	77.5%
	May	34188	9726	43914	22.1%	77.9%
	June	29968	8834	38802	22.8%	77.2%
	July	31173	7938	39111	20.3%	79.7%
	Aug	36915	9050	45965	19.7%	80.3%
	Sep	32447	7493	39940	18.8%	81.2%
	Oct	32491	6971	39462	17.7%	82.3%
	Nov	35874	7274	43148	16.9%	83.1%
	Dec	22845	5505	28350	19.4%	80.6%
2024	Jan	23317	6322	29639	21.3%	78.7%
	Feb	34595	7110	41705	17.0%	83.0%
	Mar	33861	7028	40889	17.2%	82.8%
	April	31537	7007	38544	18.2%	81.8%
	May	35465	7778	43243	18.0%	82.0%
	June	29631	6578	36209	18.2%	81.8%
	July	35059	7569	42628	17.8%	82.2%
	Aug	35293	7800	43093	18.1%	81.9%

	Sep	33736	7221	40957	17.6%	82.4%
	Oct	34784	7445	42229	17.6%	82.4%
	Nov	33798	7275	41073	17.7%	82.3%
	Dec	22860	5614	28474	19.7%	80.3%
